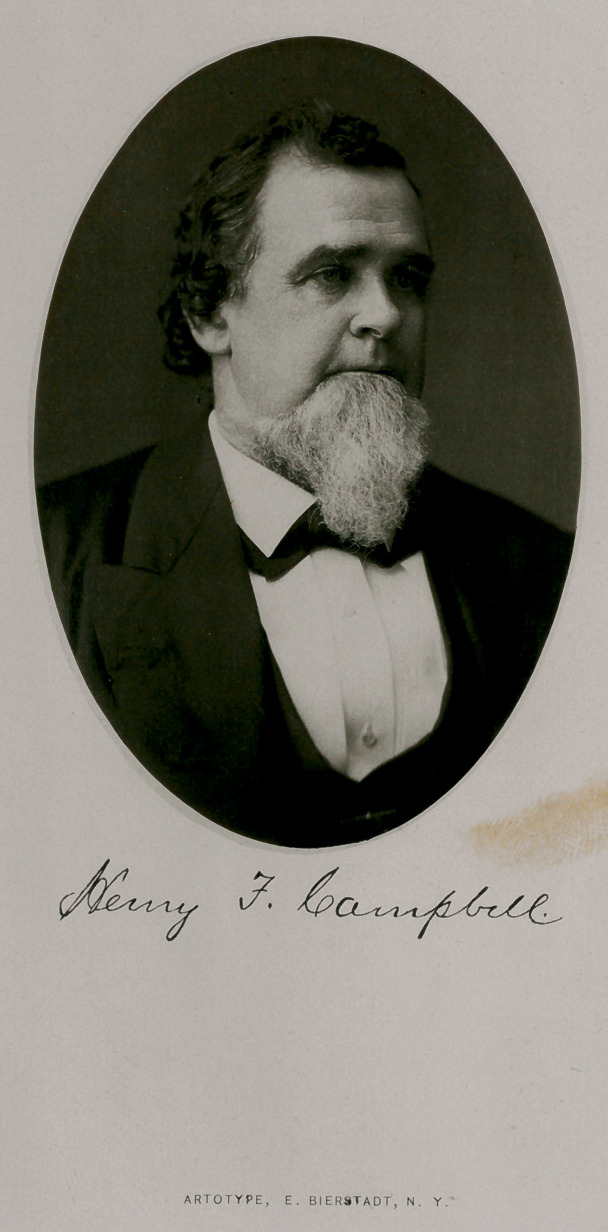# Henry Frazier Campbell, M. D.

**Published:** 1884-09

**Authors:** 


Henry F. Campbell

ARTOTYPE, E. BIERSTADT, N. Y.

giving responsible advertisers a notice when you have that notice properly displayed, that readers may not be misled; and feeling that way, we shall continue to give advertisers such notices as they deserve.

---

### HENRY FRAZER CAMPBELL, M. D.

The subject of this sketch was born in Savannah, Ga., February 10th, 1824. His father, James Colgan Campbell, was born in County Antrim, Ireland. His mother was Mary R. (Eve) Campbell, the only daughter of Joseph Eve, and a sister of Dr. Joseph A. Eve, of Augusta. Dr. H F. Campbell, after having received an academic education, began the study of medicine at the age of fifteen, and in March, 1842, when eighteen years of age, was graduated M. D. from the Medical College of Georgia—now the Medical Department of the University of Georgia. Immediately after graduating in medicine he established himself in the practice of his profession in Augusta, Ga., where he has continued to reside and practice his profession, with an exception of the period of time from 1862 to 1865, when he was engaged in the military service of the Confederate States at Richmond, Va.; and 1867-'68 when he resided in New Orleans, La., and filled the chair of Professor of Surgery in the "New Orleans School of Medicine."

Francis Bacon said: "I hold every man a debtor to his profession; from the which as men of course do seek to receive countenance and profit, so ought they of duty to endeavor themselves by way of amends to be a help and ornament thereunto." Let us inquire how Dr. Campbell has discharged this duty to his profession.

From 1842 to 1854 he filled the position of Assistant Demonstrator and Demonstrator of Anatomy in the Medical College of Georgia; from 1854 to 1857 was Professor of Comparative and Microscopical Anatomy, 1857-'67 was Professor of Anatomy; in 1867-'68 was Professor of Surgery in New Orleans School of Medicine, and Clinical

Lecturer in Charity Hospital, New Orleans, La. In the fall of 1868 the Medical College of Georgia created the chair of Operative Surgery and invited Dr. Campbell to return from New Orleans to Augusta and accept the Professorship thus created. Dr. Campbell complied with the request and filled the Chair of Operative Surgery and Gynæcology until 1881 when Dr. L A. Dugas resigned the Chair of Principles and Practice of Surgery, and Dr Campbell was elected Professor of Principles and Practice of Surgery and Gynæcology.

During the late war he was Medical Director of the Georgia Military Hospitals at Richmond, Va., and a member of the Army Medical Examining Board of the Confederate States; he is a member of the American Medical Association, was Vice-President in 1858 and is now its President, elected at the late meeting in Washington, D C.; member of Medical Association of Georgia and elected its President in 1871; a member of the American Public Health Association, and Vice-President in 1880; a member and one of the founders of the American Gynæcological Society; member and Vice-President of the American Surgical Association; President Augusta Library and Medical Society in 1878; a correspondent of the Academy of Natural Sciences of Philadelphia, elected 1858; corresponding member of the Imperial Academy of Medicine of St. Petersburg, Russia, elected 1860; elected in 1878 Foreign Corresponding Member of the Medical Society of Sweden; member of State Board of Health of Georgia; elected member of Abingdon Academy of Medicine in 1879; elected in 1882 an honorary member of the American Academy of Medicine.

The following list presents most of the professional contributions from the pen of Dr. Campbell, *i. e*: "Abortive Treatment of Gonorrhœa," *Southern Medical and Surgical Journal*, January, 1845; "Abuse of Diuretics," *ibid.*, 1845; "Observations on Cutaneous Diseases," ibid, August and October, 1845, and August, 1847; "Infantile Paroxysmal Convulsions, their Identity with Intermittent Fever, and their Treatment with Quinine," *ibid.*, October, 1849; "Dentition in Producing Disease (Reflex-secretory or Vaso-motor Action),"

*ibid.*, June, 1850; "Epidemic Dengue Fever," etc., *ibid.*, January, 1851; "Law Governing the Distribution of Striped and Unstriped Muscular Fibre," *ibid.*, March, 1851, and Transactions American Medical Association, vol. iv; "Injuries to the Cranium in their Relation to Consciousness," *Southern Medical and Surgical Journal*, 1851; "Bilateral Lithotomy," *ibid.*, August, 1851; "Unusual Form of Fever and Dysentery," *ibid.*, 1851; "Report on Surgery," Transactions Medical Association of Georgia, 1852; "The Nature of Typhoidal Fever," etc., Transactions American Medical Association, May, 1853; "The Sympathetic Nerve in Reflex Phenomena a Question of Priority of Announcement with M. Claude Bernard," etc., *ibid.*, May, 1853; "Strangulated Ventral Hernia during Pregnancy," *Southern Medical and Surgical Journal*, January and March, 1857; "Clinical Lecture on Traumatic Tetanus," *ibid.*, February, 1857; "Meckel's Ganglion," etc., *Southern Medical and Surgical Journal*, February, 1858; "Classification of Febrile Diseases by the Nervous System," Transactions American Medical Association, 1857; "The Nervous System in Febrile Diseases, Excito-secretory or Reflex 'Vaso-motor' Action, the Basis of their Phenomena," *ibid*, 1858; "The Secretory and Excito-secretory System," 1 vol., 8 vo., pp. 135, Lippincott, Philadelphia, 1858; "Caffeine as an Antidote to Opium," *Southern Medical and Surgical Journal*, May, 1860; "A New 'Ready Method,' Artificial Respiration in the Sitting Posture," *ibid*, May, 1860; "Croup, a Paroxysmal Neurosis, Its Treatment with Quinine," *ibid*, 1860; "The Effect of Caffeine upon the Muscular System," *ibid*, 1860; "The Georgia Military Hospitals of Richmond," pamphlet, Augusta, Ga, 1861; "Traumatic Hemorrhage and the Arteries," etc., a chapter in the 'Confederate Manual of Military Surgery,' 1 vol., 12 mo., pp. 297, Richmond, 1863; (in this chapter the principle of ligating the main arterial trunk of a limb, for the cure of inflammation, and for gangrene, is announced); "The Hunterian Ligation of Arteries in Destructive Inflammation," *Southern Journal of the Medical Sciences*, New Orleans, August, 1866; "Inflammation," Cooper's Surgical Dictionary, London, 1872; "Position, Pneumatic Pressure, and Mechanical Appliance in Uterine Displacements," etc., *Atlanta Medical and Surgical Journal*, June, 1875; "Registration and Sanitation," first Report State Board of Health of Georgia, 1875; "Blood-Letting in Puerperal Eclampsia," etc., *American Journal of Obstetrics and Diseases of Women and Children*, August, 1876; "Railroad Transportation of Disease Germs," etc, (Yellow and Dengue Fever in the South in 1839, 1850, 1854 and 1876) Report of State Board of Health of Georgia, 1876; "Pneumatic Self-replacement in Dislocations, of the Gravid and Non-Gravid Uterus," Translations American Gynæcological Society, 1875; "Calculi in the Bladder after the Cure of Vesico-vaginal Fistula," *ibid*, 1876; "The Neuro-Dynamic Etiology and Pathology of Urinary Calculus," read before the Surgical Section of the International Medical Congress, 1876; "Arterial Ligation in the Treatment of Traumatic Inflammation and Gangrene," read before International Medical Congress, 1876; Strictures of the Œsophagus their Nature and Treatment," translations American Surgical Association, vol. I., 1883; "Rectal Alimentation in the Nausea and Inanition of Pregnancy," Transactions American Gynæcological Society. Any sketch which omitted to mention the discovery of the Excito-secretory System of Nerves by Dr. Campbell would do him great injustice. Three years after his discovery the great English physiologist, Marshall Hall, announced through the *London Lancet* that he had discovered this system. Upon reading Dr. Hall's paper Dr. Campbell promptly presented him with copies of his publications several years preceding that of Dr. Hall. Dr. Hall immediately, by letter to Dr. Campbell and through the medical press, withdrew his claim and awarded the credit to Dr. Campbell. He said: "It would be unjust to deny that Dr. Campbell has the merit of having first called attention to the excito-secretory system, in the year 1850, and that he imposed this very designation in 1853. So far, Dr. Campbell's claims are undeniable, and we would say *palmam qui meruit ferat*." An examination of the vast number of contributions which he has made to medical science attests the versatility of his genius, and points to the enthusiasm of the scientist.

The subject of this sketch is one of the most renowned physicians in America and his writings are quoted in European as well as American text-books—several of them having been translated into other languages. He has an international as well as national reputation in the diverse fields of Physiology, Gynæcology, Surgery and Sanitation.

As a leader in medicine Dr. Campbell has few equals—no superiors: he is possessed of a wonderful memory, and never refers to notes in lecturing; he is gifted with smoothness of speech, charming and elegant diction, complete with personal magnetism and the ability to impart instruction to his students in the most acceptable manner. In the social sphere he is one of the most entertaining of men. He is fully qualified (as he sometimes does) to entertain his friends with classical and poetical citations, and discussions of the higher order of novels, questions of science, political or church history. He is warm-hearted, genial, proverbially good-natured.

> " A merrier man,
> Within the limit of becoming mirth,
> I never spent an hour's talk withal.

In religion Dr. Campbell—Scotch-Irish as he is—is a Presbyterian. He has for a number of years been an elder in the First Presbyterian Church of Augusta.

As a physician he is tender and gentle as a woman, inspiring the sick and afflicted with kind and hopeful words, and ministering with considerate attention and signal ability to their needs. No physician possesses the confidence and love of his patients to a greater extent than he. He has for a number of years withdrawn as far as possible from the general practice of medicine, and endeavored to confine his practice to Surgery and Gynæcology. In these two fields he has the largest and most lucrative practice of any physician in his section of country. His practice is largely a consultation one—he being frequently called to all sections of Georgia and South Carolina.

Dr. Campbell's genius, wonderful work, and self-sacrificing devotion to the science of medicine have secured for him an acknowledged high position in the medical profession. The many tokens of esteem and honor which have been conferred upon him by his professional brethren have been fully merited. In his recent election to the presidency of the American Medical Association—the very highest honor which can be accorded an American physician – his professional brethren of the United States have in honoring him honored themselves. Let him who has justly won it bear the palm.

## Figures and Tables

**Figure f1:**